# Retinitis associated with double infection of Epstein–Barr virus and varicella-zoster virus: A case report

**DOI:** 10.1097/MD.0000000000011663

**Published:** 2018-08-03

**Authors:** Tomohito Sato, Riki Kitamura, Toshikatsu Kaburaki, Masaru Takeuchi

**Affiliations:** a Department of Ophthalmology, National Defense Medical College, Saitama; b Department of Ophthalmology, The University of Tokyo, Bunkyo, Tokyo, Japan.

**Keywords:** double infection, Epstein–Barr virus, human herpesvirus, retinitis, varicella-zoster virus

## Abstract

**Rationale:**

Chronic uveitis with immunosuppressive agents could develop chronic herpetic retinitis with varicella-zoster virus (VZV) or herpes simplex virus (HSV). Ocular Epstein–Barr virus (EBV) infection develops uveitis and vitritis, but the clinical feature of EBV retinitis is not typical as a viral retinitis. EBV retinitis is rare, and only a few cases of EBV retinitis have been reported. Herein, we describe a case of retinitis with EBV and VZV which were the primary viruses verified by multiplex polymerase chain reaction (PCR).

**Patient concerns:**

A 75-year-old woman suffered from sudden visual loss in the left eye. She had been diagnosed with rheumatoid arthritis. At presentation, visual acuity (VA) was 20/400 in the left eye. Slit lamp examination disclosed fine white keratic precipitates with infiltrating cells and dense vitreous opacities in the anterior segment and vitreous. Fundus photographs showed multifocal chorioretinal scars in macula and peripheral retina, and granular lesions surrounding arcade vessels.

**Diagnoses:**

Ocular toxoplasmosis was primarily suspected.

**Interventions:**

However, serological test showed negative of toxoplasmosis. Therefore, a diagnostic and therapeutic vitrectomy was performed. Vitreous fluid sample was used for multiplex PCR for detection of human herpesvirus (HHV) -1 to -8, toxoplasmosis and toxocariasis.

**Outcomes:**

Multiplex PCR detected 5.8 × 10^5^ copies/mL of EBV-deoxyribonucleic acid (DNA), and 3.6 × 10^6^ copies/mL of VZV-DNA in the sample. Therefore, we could diagnose the unidentified panuveitis a retinitis associated with double infection of EBV and VZV. At 85 days after the vitrectomy, VA of the left eye recovered to be 20/16.

**Lessons:**

Elderly patients under immunosuppression may be susceptible to develop retinitis associated with infection of multiple HHVs, and multiplex PCR is an excellent tool to diagnose an unidentified panuveitis resembling this case.

## 1 Introduction

Herpetic retinitis is an uncommon, but vision-threatening infection.^[1]^ Chronic uveitis with immunosuppressive agents could develop chronic herpetic retinitis with varicella-zoster virus (VZV) or herpes simplex virus (HSV) type 2 detected in ocular samples.^[2]^ Epstein–Barr virus (EBV) known as human herpesvirus (HHV) 4 is transmitted by oral secretions, infects more than 90% of humans, and is usually carried lifelong as an asymptomatic infection.^[3–5]^ Ocular EBV infection was described as uveitis, vitritis and optic disk vasculitis, but the clinical feature of EBV retinitis is not typical as a viral retinitis.^[5]^ EBV retinitis is rare, and only a few cases of EBV retinitis have been reported.^[5–7]^ To the best of our knowledge, there is no report of EBV retinitis with VZV detected in the vitreous fluid. Herein, we describe a case of retinitis with EBV and VZV which were the primary viruses verified by multiplex polymerase chain reaction (PCR).

## 2 Case report

**Figure 1:**
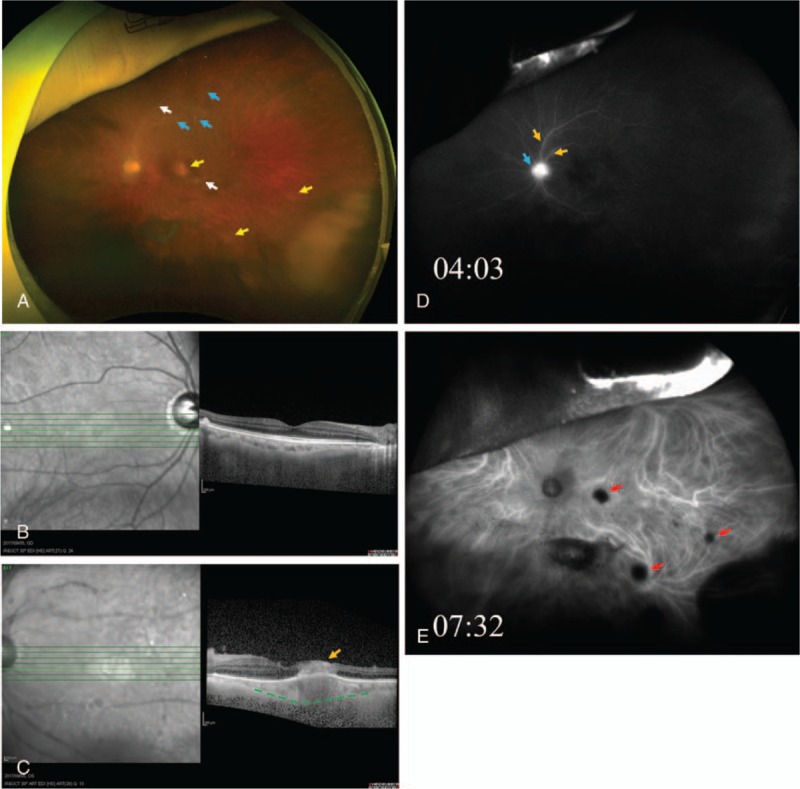
Fundus findings at initial visit. Fundus images showed chorioretinal scars without pigmentation in macula and peripheral retina (yellow allows), and white sheathing vessels at the posterior pole (white allows), and granular lesions (blue allows). (A) OCT demonstrated normal retina in the right eye (B), but revealed retinal edema in all retinal layers (orange allow) and choroidal thickness in the lesion of retinal edema (green dotted line) in the left eye. (C) FA images showed leakage from retinal arcade vessels (orange allows), and hyperfluorescece at the optic disc (blue allow) in the early phase. (D) ICGA images demonstrated multifocal lesions with filling defects correlated with chorioretinal scars (red allows) through the early, middle and late phases (E). FA = fluorescein angiograms, ICGA = indocyanine green angiograms, OCT = optical coherence tomography.

**Figure 2:**
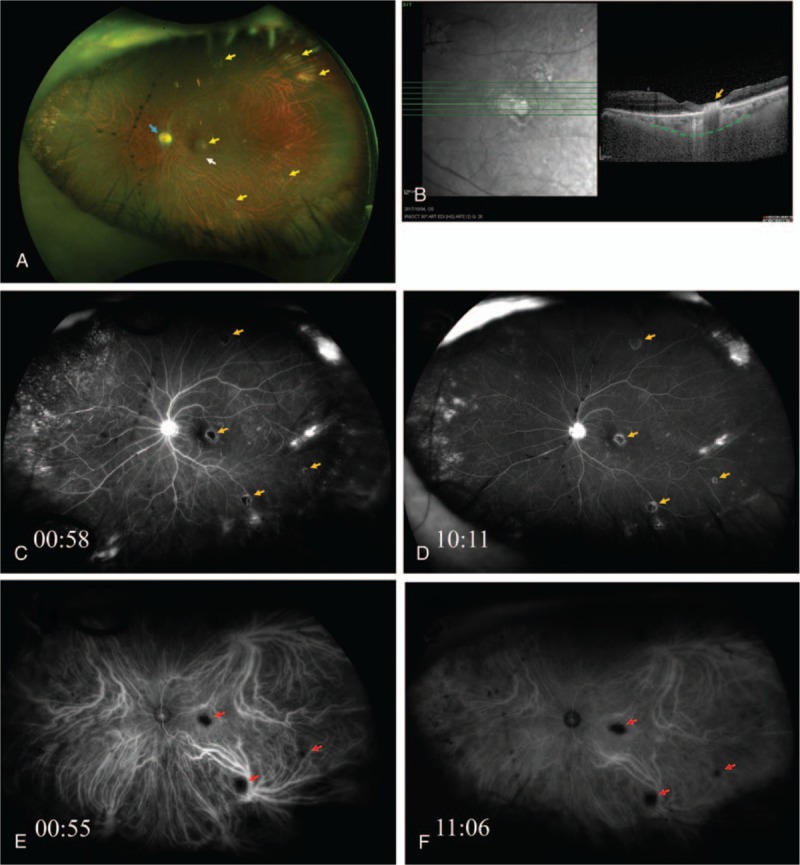
Fundus findings at 9 days after vitrectomy in the left eye. Fundus photographs demonstrated multifocal scar lesions without pigmentation (yellow allows), white sheathing vessel (white allow), and pallor of the optic disc (blue allow). (A) OCT demonstrated atrophic thinning retia in all retinal layers (orange allow), and choroidal thickness in the lesion of chorioretinal scar (green dotted line). (B) FA and ICGA images showed filling defects in the multifocal chorioretinal scars (FA; orange allows, ICGA; red allows) through the early, middle and late phases (C to F). FA = fluorescein angiograms, ICGA = indocyanine green angiograms, OCT = optical coherence tomography.

This study protocol was not approved by the Ethics Committee of National Defense Medical College as it was not deemed necessary, this being a retrospective case report. The Declaration of Helsinki was followed in this case report. Patient consent has been obtained for the publication of the contents in this report. A 75-year-old woman visited a local eye clinic because of sudden visual loss in the left eye. She was diagnosed with an unidentified panuveitis, and referred to our hospital. As the past history, she had been diagnosed with rheumatoid arthritis and prescribed with prednisolone 4 mg/day and methotrexate 12 mg/week by oral administration for 7 years, and had been sick due to summer heat. At the initial examination, the best-corrected visual acuity (BCVA) was 20/30 in the right eye and 20/400 in the left eye. Intraocular pressure was 11.5 mm Hg in the right eye and 12.5 mm Hg in the left eye. Slit lamp examination disclosed fine white keratic precipitates (KP) with infiltrating cells and dense vitreous opacities in the anterior segment and vitreous of left eye. Fundus photographs demonstrated multifocal chorioretinal scars without pigmentation in the macula and peripheral retina of left eye, white sheathing vessels at the posterior pole, and granular lesions surrounding superior temporal retinal arcade vessels (Fig. 1A) although it was obscured by vitreous opacity. In the fellow eye, there was no abnormal finding in the fundus (Fig. 1B). Spectral-domain optical coherence tomography (SD-OCT) revealed retinal edema in all retinal layers, and choroidal thickness in the lesion of retinal edema (Fig. 1C). Fluorescein angiography (FA) images showed leakage of the dye from superior temporal retinal arcade vessels, and hyperfluorescece at the optic disc in the early phase (Fig. 1D). Indocyanine green angiography (ICGA) images depicted multifocal lesions with filling defects of the dye which were correlated with multifocal chorioretinal scars throughout the early, middle, and late phases (Fig. 1E). Since ocular toxoplasmosis was primarily suspected from the ocular findings, albendazole 600 mg/day by oral administration and 0.1% betamethasone eye drops 4 times/day were initiated, and doses of systemic corticosteroids were increased from prednisolone 4 mg/day to 30 mg/day. However, the inflammatory symptoms deteriorated. Furthermore, serological tests for toxoplasmosis and toxocariasis were negative, and complement fixation antibody titer of cytomegalovirus, VZV or HSV was 128 times, 16 times or 4 times, respectively. Therefore, a diagnostic and therapeutic vitrectomy was performed. Vitreous fluid sample was collected and used for cultures of bacteria and fungus, as well as multiplex PCR for detection of HHV-1 to -8, bacterial 16S ribosomal ribonucleic acid (rRNA), fungus 28S rRNA, syphilis, tuberculosis, toxoplasma and toxocariasis.^[8]^ Multiplex PCR detected 5.8 × 10^5^ copies/mL of EBV-deoxyribonucleic acid (DNA), and 3.6 × 10^6^ copies/mL of VZV-DNA in the sample. Thus, we could diagnose that the unidentified panuveitis with multifocal chorioretinal scars was a retinitis associated with double infection of EBV and VZV. After the vitrectomy, the panuveitis became under control without any systemic administrations of antibiotics, antiviral agents and corticosteroids. At 9 days after the vitrectomy, slit-lamp examination showed elimination of KP and few infiltrating cells in the anterior chamber. Fundus photographs demonstrated multifocal lesions of yellowish white chorioretinal scars without pigmentation, white sheathing vessels in the macula, and pallor of the optic disc (Fig. 2A). SD-OCT demonstrated atrophic thinning retia in all retinal layers, and choroidal thickness under the lesion of chorioretinal scar in the macula (Fig. 2B). FA and ICGA images depicted filling defects of the dyes in the multifocal chorioretinal scars throughout the early, middle, and late phases (Fig. 2C–F). At 85 days after the vitrectomy, BCVA of the left eye recovered to be 20/16.

## 3 Discussion

The clinical features of KP with infiltrating cells, vitreous opacities and retinitis are common in panuveitis, but primary panuveitis with chorioretinal scar juxta macula is limited to several diseases such as ocular toxoplasmosis, ocular toxocariasis and multifocal choroiditis and panuveits (MCP). Ocular toxoplasmosis has distinctive clinical findings, that is, an area of active necrotizing retinochoroiditis at the edge of a pigmented chorioretinal scar.^[9]^ At first, we suspected the present case a panuveitis associated with ocular toxoplasmosis from the ocular findings. But, serological test revealed negative of toxoplasmosis infection, and antiparasitic medication was also ineffective. Therefore, we supposed that this case was not ocular toxoplasmosis but an unidentified panuveitis with multifocal chorioretinal scars, and could diagnose the case a retinitis associated with double infection of EBV and VZV using a multiplex PCR.

The clinical features of ocular EBV infection remains unclear although 90% of adults aged between 35 and 40 years have been infected.^[3,5]^ It has been reported that healthy patients with MCP showed serological evidence of recent or continuing chronic EBV infection without acute infectious mononucleosis-like illnesses.^[10]^ Therefore, we suppose that EBV retinitis with MCP may account for the multifocal chorioretinal scars in our case. Furthermore, the granular lesions surrounding superior temporal retinal arcade vessels were something that made us suspect a chronic uveitis. In the past clinical course, treatment of rheumatoid arthritis with systemic corticosteroids and immunosuppressive agents as well as physical weakness due to the summer heat may have resulted in a substantially immunocompromised state. Under the immunosuppressive condition, the chronic uveitis could have developed simultaneously with EBV retinitis.

Acute retinal necrosis (ARN) syndrome is a rare infectious viral uveitis syndrome that manifests as a necrotizing retinitis, and may result in a devastating visual outcome if not accurately diagnosed and treated.^[11]^ Multiple members of HHV family including VZV, HSV-type 1 and -type 2 have been recognized as pathogenic agents of ARN syndrome.^[12,13]^ In clinical features, ARN syndrome is characterized by anterior uveitis, vitritis, and patchy or confluent lesions of white- or cream-colored areas of necrotizing retinitis that rapidly extend posteriorly from the peripheral retina.^[11,14]^ Occlusive vasculitis also involves the retinal and choroidal vasculature.^[15]^ ARN syndrome is observed most commonly in healthy patient but occasionally occurs in immunocompromised host.^[16,17]^ In our case, there was no characteristic finding of ARN syndrome such as patchy and confluent lesions of white- or cream-colored necrotizing retinitis in the peripheral retina. However, since multifocal chorioretinal scars without pigmentation, white sheathing vessel and pallor of the optic disc were observed, this case was diagnosed as a chronic uveitis with double infection of EBV and VZV instead of ARN syndrome.

Diagnostic and therapeutic vitrectomy greatly assists in the diagnosis and guide alternative management strategies for infectious uveitis.^[18]^ Furthermore, the vitrectomy could remove the vitreous with accumulations of inflammatory factors and etiologic agents. In general, oral and intravenous acyclovir is effective in treating initial and recurrent herpes simplex and varicella-zoster virus infections.^[19]^ The selective antiherpetic activity of acyclovir is due to the inhibition of the herpes-specified DNA polymerase.^[20–23]^ In other words, the drug efficacy of acyclovir is not elimination of infected cells and HHVs, but inhibition of HHVs proliferation. In our patient, there was no need to perform any systemic administrations of antibiotics, antiviral agents and corticosteroids after the vitrectomy. Therefore, we speculate that the vitrectomy reduced the amounts of EBV and VZV in the vitreous similar to the inhibition of HHVs proliferation by antiviral agents, and could improve the condition of intraocular inflammation.

In conclusion, the present case suggests that elderly patients under immunosuppression may be susceptible to develop retinitis associated with infection of multiple HHVs, and supports that multiplex PCR is an excellent tool to diagnose an unidentified panuveitis. Infections uveitis similar to the present case is likely to increase in the future of super-aging society.

## Author contributions

**Data curation:** Riki Kitamura.

**Formal analysis:** Toshikatsu Kaburaki.

**Funding acquisition:** Masaru Takeuchi.

**Investigation:** Tomohito Sato, Riki Kitamura, Toshikatsu Kaburaki.

**Writing ± original draft:** Tomohito Sato.

**Writing ± review & editing:** Masaru Takeuchi.
